# Distinct Roles of Soluble and Transmembrane Adenylyl Cyclases in the Regulation of Flagellar Motility in *Ciona* Sperm

**DOI:** 10.3390/ijms150813192

**Published:** 2014-07-28

**Authors:** Kogiku Shiba, Kazuo Inaba

**Affiliations:** Shimoda Marine Research Center, University of Tsukuba, Shimoda 5-10-1, Shizuoka 415-0025, Japan; E-Mail: kogiku@kurofune.shimoda.tsukuba.ac.jp

**Keywords:** calcium, sperm chemotaxis, fertilization, protein kinase, protein phosphorylation

## Abstract

Adenylyl cyclase (AC) is a key enzyme that synthesizes cyclic AMP (cAMP) at the onset of the signaling pathway to activate sperm motility. Here, we showed that both transmembrane AC (tmAC) and soluble AC (sAC) are distinctly involved in the regulation of sperm motility in the ascidian *Ciona intestinalis*. A tmAC inhibitor blocked both cAMP synthesis and the activation of sperm motility induced by the egg factor sperm activating and attracting factor (SAAF), as well as those induced by theophylline, an inhibitor of phoshodiesterase. It also significantly inhibited cAMP-dependent phosphorylation of a set of proteins at motility activation. On the other hand, a sAC inhibitor does not affect on SAAF-induced transient increase of cAMP, motility activation or protein phosphorylation, but it reduced swimming velocity to half in theophylline-induced sperm. A sAC inhibitor KH-7 induced circular swimming trajectory with smaller diameter and significantly suppressed chemotaxis of sperm to SAAF. These results suggest that tmAC is involved in the basic mechanism for motility activation through cAMP-dependent protein phosphorylation, whereas sAC plays distinct roles in increase of flagellar beat frequency and in the Ca^2+^-dependent chemotactic movement of sperm.

## 1. Introduction

In most animal species, sperm are immotile in testis or sperm duct. Flagellar motility is activated and regulated by external cues such as specific ions, osmotic change or factors from the egg or female genital tract at fertilization [1]. In the ascidian *Ciona intestinalis* and *C. savignyi*, a sulfated steroid called sperm activating and attracting factor (SAAF) induces both sperm motility activation and chemotaxis [2]. Cyclic AMP (cAMP) is one of the most important intracellular factors in the signaling pathway for SAAF-induced sperm activation. A 21 kDa light chain of outer arm dynein (LC2) and a 26 kDa axonemal protein are phosphorylated, and dynein intermediate chains IC2 and IC116 are dephosphorylated in a cAMP-dependent manner, resulting in activation of flagellar motility [3,4].

Adenylyl cyclases (ACs) are the key enzyme that synthesizes cAMP at the onset of the signaling pathway to activate axonemal dyneins through protein phosphorylation. In sea urchin, frog and mammalian sperm, both soluble adenylyl cyclase (sAC) and transmembrane adenylyl cyclases (tmACs) are present and play important roles in sperm function [5,6,7,8,9,10]. However, the roles of two types of ACs are controversial. tmACs are suggested to be involved in the regulation of sperm motility, in particular during chemotactic behavior [11,12]. However, tmACs are shown localized at the acrosome [9,13]. sAC-deficient mice are infertile, lacking sperm motility [8]. In fact, sAC is localized in sperm flagella in mouse [8] and sea urchin [14]. In this study we first characterized sperm ACs in the ascidian *C. intestinalis*. Using inhibitors specific to each type of AC, we demonstrate that tmAC or sAC critically plays a distinct role in the basic activation of flagellar motility or increase in flagellar beat frequency and Ca^2+^-dependent regulation of flagellar waveform during chemotactic movement, respectively.

## 2. Results and Discussion

### 2.1. Expression of Adenylyl Cyclase (AC) Genes in Ciona Testis

**Figure 1:**
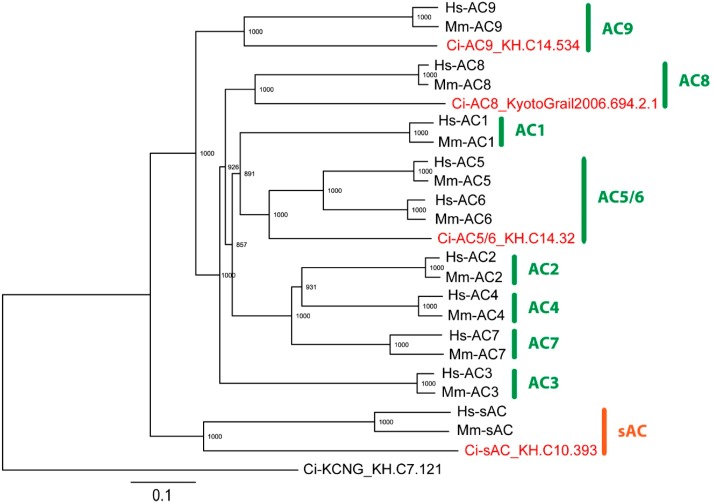
Phylogenetic analysis of adenylyl cyclases. *Ciona* tetrameric potassium-selective cyclic nucleotide-gated channel (Ci-KCNG) was used as the outgroup. The value on each branch represents the number of times that a node was supported in 1000 bootstrap pseudo replications. BLASTP search of adenylyl cyclase (AC) against *Ciona* genome revealed four AC gene models. Phylogenetic analysis suggests that ACs encoded by these four genes are grouped with mammalian AC5/6, AC8, AC9 and soluble AC (sAC) respectively. Accession numbers of proteins are: *Homo sapiens*: Hs-AC1 (NP_066939), Hs-AC2 (NP_065433), Hs-AC3 (NP_004027), Hs-AC4 (NP_640340), Hs-AC5 (NP_899200), Hs-AC6 (NP_056085), Hs-AC7 (NP_001105), Hs-AC8 (NP_001106), Hs-AC9 (NP_001107), Hs-sAC (NP_060887); *Mus musculus*: Mm-AC1 (NP_033752), Mm-AC2 (NP_705762), Mm-AC3 (NP_001153008), Mm-AC4 (NP_536683), Mm-AC5 (NP_001012783), Mm-AC6 (EDL04180), Mm-AC7 (NP_001032813), Mm-AC8 (NP_033753), Mm-AC9 (NP_033754), Mm-sAC (NP_766617). Sequences of *Ciona* proteins are obtained from the Ghost database (http://ghost.zool.kyoto-u.ac.jp). Proteins encoded by *Ciona* gene models are indicated in red. The bars in green or in red represents tmAC or sAC, respectively.

**Figure 2:**
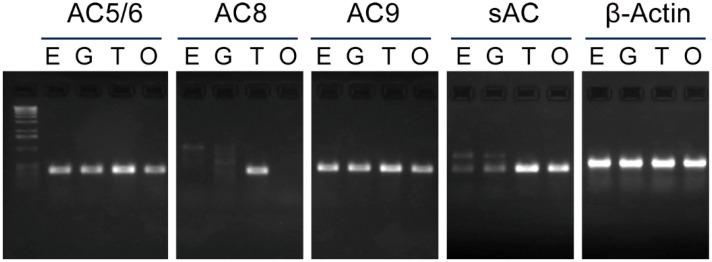
Tissue expression patterns of the *adenylyl cyclase* (*AC*) genes in *Ciona* tissues. RT-PCR analysis of mRNA from several adult *Ciona* tissues shows that genes encoding all AC isoforms are expressed in testis but the expression of *AC8* is testis-specific. Expression of *sAC* gene was found in testis and ovary. β-actin was used as an internal control. E, endostyle; G, gill; T, testis; and O, ovary.

Ten isoforms of AC have been cloned and characterized in mammals; nine are tmACs and the other is sAC [5,6]. Their distributions and regulations differ among isoforms. To clarify the functions of AC in *Ciona* sperm, we first examined the expression of AC genes in *Ciona* tesits. A search for AC genes in the genome of *Ciona intestinalis* revealed the presence of three tmAC genes and one sAC gene. Phylogenetic analysis suggested that three tmAC genes are grouped with mammalian AC5/6, AC8 and AC9 (Figure 1). Next, we examined the expression of AC genes by reverse-transcriptase PCR (RT-PCR) using PCR primers for these four AC genes. Recently, AC5/6 was reported to be expressed in the intestine of *Ciona* juvenile [15]. From the analysis in the present study, it turned out that all AC isoforms were expressed in testis but the expression of AC8 is only testis-specific. sAC was expressed in testis at a high level but was also significantly expressed in ovary (Figure 2).

### 2.2. Effects of AC Inhibitors on the Motility Activation of Ciona Sperm

#### 2.2.1. Sperm Activating and Attracting Factor (SAAF)-Induced Motility Activation

**Figure 3:**
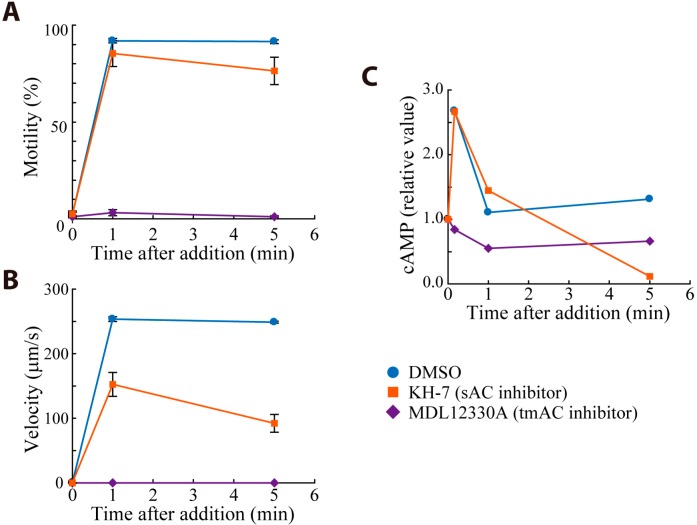
Effects of AC inhibitors on sperm activating and attracting factor (SAAF)-induced activation of *Ciona* sperm motility and cAMP synthesis. The percentage of motile sperm (**A**); curvilinear velocity (**B**) and intracellular cAMP level (**C**) were measured before, 1 min and 5 min after the addition of 100 nM SAAF. Sperm were treated with 0.5% dimethyl sulfoxide (DMSO, control), 100 μM MDL12330A or 10 μM KH-7 respectively for 3 min before SAAF addition. Values are means ± standard error (SE) of the results from three experiments.

*Ciona* sperm show less motility in seawater but become activated by an egg-derived factor, sperm activating and attracting factor (SAAF) [16]. SAAF induces Ca^2+^ influx, membrane hyperpolarization and activation of adenylyl cyclase to produce cAMP [16]. Transient increase of intracellular cAMP and subsequent cAMP-dependent phosphorylation of dynein subunits are observed in response to the addition of SAAF [3,17]. Thus, cAMP is one of important factors for SAAF-induced sperm motility activation. To specify an AC isoform that is involved in SAAF-induced motility activation, we examined the effect of AC inhibitors specific to tmAC or sAC on motility activation and cAMP synthesis (Figure 3). The tmAC inhibitor, MDL12330A, completely blocked SAAF-induced motility activation and transient increase of cAMP. MDL12339A did not affect any changes on the motility Triton X-100-demembranated sperm (data not shown). In contrast, the sAC inhibitor, KH-7, did not inhibit SAAF-induced motility activation or transient increase of cAMP synthesis. However it reduced swimming velocity to half of the control and brought basal cAMP level to almost zero in 5 min. Swimming velocity is closely related to the flagellar beat frequency [18]. These results indicate that tmAC has a major role in the signaling pathway in SAAF-induced motility activation and that sAC is involved in the increase in beat frequency and in the maintenance of basal level of cAMP. This agrees with the observation in mouse sperm that a sAC activator HCO_3_^−^ induces the increase of flagellar beat frequency through cAMP-dependent protein kinase (protein kinase A; PKA) [19].

#### 2.2.2. Valinomycin- and Theophylline-Induced Motility Activation

**Figure 4:**
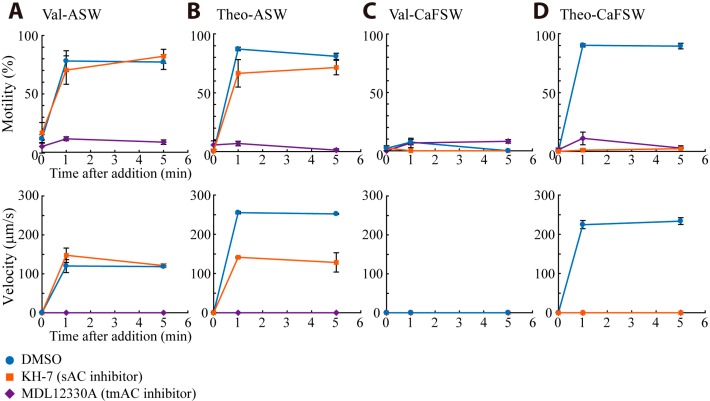
Effects of AC inhibitors on valinomycin (Val)- or theophylline (Theo)-induced activation of *Ciona* sperm motility. The percentage of motile sperm and curvilinear velocity were measured before, 1 and 5 min after the addition of 10 nM valinomycin or 1 mM theophylline in artificial sea water (ASW; **A**,**B**) or Ca^2+^-free sea water (CaFSW; **C**,**D**). Sperm were treated with 0.5% DMSO (control), 100 μM MDL12330A or 10 μM KH-7 respectively for 3 min before the addition of valinomycin or theophylline. Values are means ± SE of the results from three experiments.

Valinomycin is a potassium ion-selective ionophore and induces motility activation of *Ciona* sperm by causing membrane hyperpolarization [17]. In contrast, an increase in intracellular cAMP levels was induced by a phosphodiesterase inhibitor theophylline, resulting in the motility activation [16]. To elucidate if sAC participates in SAAF-induced signaling pathway in the motility activation, we examined the effect of AC inhibitors on valinomycin- and theophylline-induced motility activation in the presence or absence of Ca^2+^ (Figure 4). Both valinomycin and theophylline activated sperm motility in ASW but valinomycin-treated sperm swam with lower velocity (Figure 4A,B). The tmAC inhibitor MDL12330A completely suppressed both motility and swimming velocity in artificial seawater (ASW) (Figure 4A,B). These results suggest the involvement of tmAC in the motility of valinomycin-treated sperm. Valinomycin-treated sperm were immotile in Ca^2+^-free seawater (CaFSW), as also previously reported [20], suggesting that Ca^2+^-influx is necessary for tmAC and motility activation (Figure 4C).

Theophylline-treated sperm showed activated in both ASW and CaFSW. The sAC inhibitor KH-7 showed ~40% suppression of the motility at 1 min but the suppression became not significant at 5 min (Figure 4B). The velocity of theophylline-treated sperm decreased to half of the control by KH-7 in ASW (Figure 4B), supporting the idea that sAC is involved in the increase of flagellar beat frequency. In contrast, theophylline-treated sperm showed motility activation even in CaFSW. The motility was suppressed by KH-7 (Figure 4D), suggesting that sAC is involved in the sperm motility in Ca^2+^-free condition. The sAC in human sperm shows synthesis of cAMP in the absence of Ca^2+^ but is activated by Ca^2+^ in a concentration-dependent manner [21]. Therefore, it is likely that the activation of sperm motility in CaFSW by theophylline is caused by basal activity of sAC in the absence of Ca^2+^. Ca^2+^-activated sAC could be involved in other process, such as regulation of intracellular pH (see 2.2.3 in the Results and Discussion Section) and sperm chemotaxis (see 2.3 in the Results and Discussion Section).

#### 2.2.3. Sperm Motility Is Activated by HCO_3_^−^ but not by Forskolin

**Figure 5:**
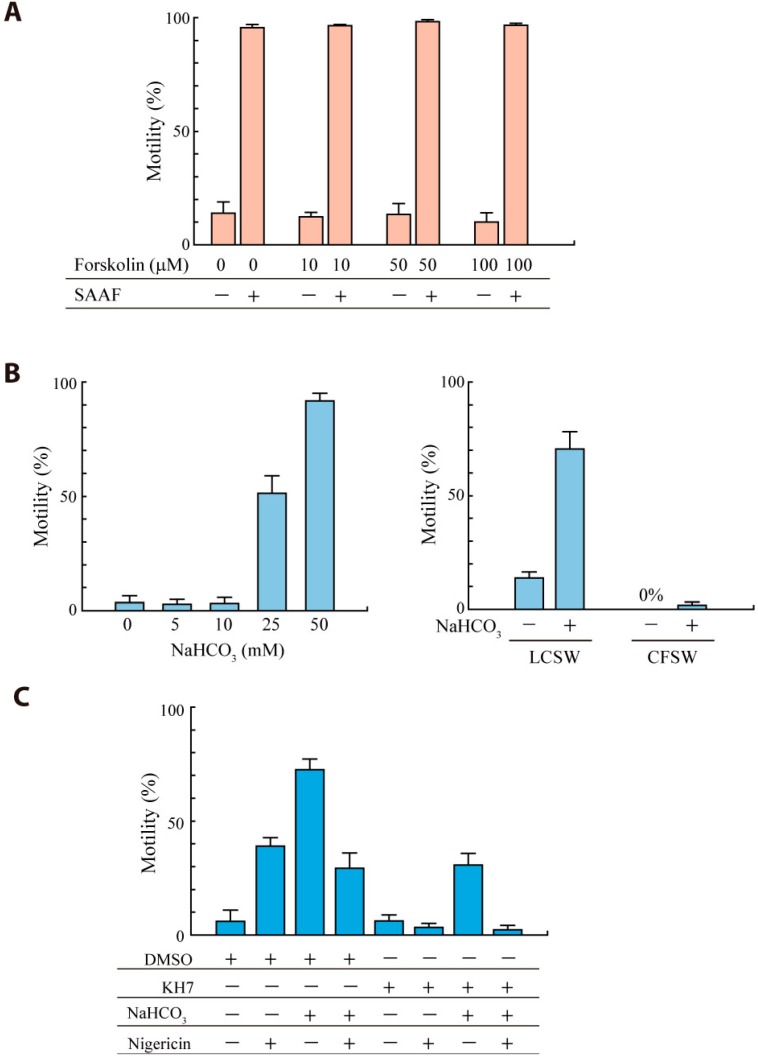
Effects of AC activators on activation of *Ciona* sperm motility. The percentage of motile sperm was measured 3 min (**A**) or 1 min (**B**,**C**) after the addition of sperm to the solutions containing forskolin (**A**), NaHCO_3_ (**B**,**C**) with or without sAC inhibitors. The concentration of KH-7 or nigericin was 10 or 5 μM, respectively. Values are means ± SE of the results from three experiments.

To further test the roles of two types of ACs in the regulation of sperm motility, we examined the effects of two activators for ACs, forskolin and HCO_3_^−^. Forskolin is a diterpene that effectively activates tmAC1 to tmAC8 [22,23] and is shown to increase cAMP and activate motility in mammalian sperm [13,24]. In *Ciona* sperm, forskolin showed no effect on the activation of sperm motility even at 100 μM (Figure 5A), suggesting that either *Ciona* tmAC5/6 or tmAC8 does not participate in SAAF-dependent activation of sperm motility.

sAC is directly activated by HCO_3_^−^ and Ca^2+^ [25,26], both of which are two important factors for capacitation of mammalian sperm [27]. Although it is known that CO_2_ inhibits motility of sperm from marine animals showing external fertilization, such as sea urchin [28,29] and flatfish [30], activation of sperm motility by HCO_3_^−^ has not been reported. Here, we observed that extracellular HCO_3_^−^ activated the motility in *Ciona* sperm (Figure 5B). Because insoluble CaCO_3_ formed by the addition of high concentration of HCO_3_^−^ into sea water disturbed the observation of sperm motility, we used the artificial sea water containing 1 mM CaCl_2_ (low calcium sea water; LCSW). The activation was observed in LCSW but not in CaFSW (Figure 5B), indicating that extracellular Ca^2+^ is required for HCO_3_^−^mediated motility activation. To see if the action of HCO_3_^−^ is due to the change in intracellular pH, we used an antiporter of H^+^ and K^+^, nigericin, to bring intracellular pH to the same pH of extracellular solution. Nigericin itself activated sperm motility in ASW at pH 7.5, but the level of activation was lower than of sperm activated by HCO_3_^−^ (Figure 5C). The motility of HCO_3_^−^-activated sperm was reduced by nigericin, suggesting that intracellular pH of HCO_3_^−^-activated sperm was higher than 7.5. KH-7 inhibited both nigericin-activated sperm and HCO_3_^−^-activated sperm. These results suggest that the action of HCO_3_^−^ is mediated by sAC in a mechanism closely coupled with the increase in intracellular pH. It is likely that the activity of *Ciona* sAC is highly dependent on pH like the case in sea urchin sperm [14] and that cAMP produced by sAC activates a Na^+^/H^+^ exchanger (sNHE) through cyclic nucleotide binding site to raise intracellular pH [31,32] (see [33] for review).

### 2.3. Effects of sAC Inhibitors on Sperm Chemotaxis and Swimming Trajectory

**Figure 6:**
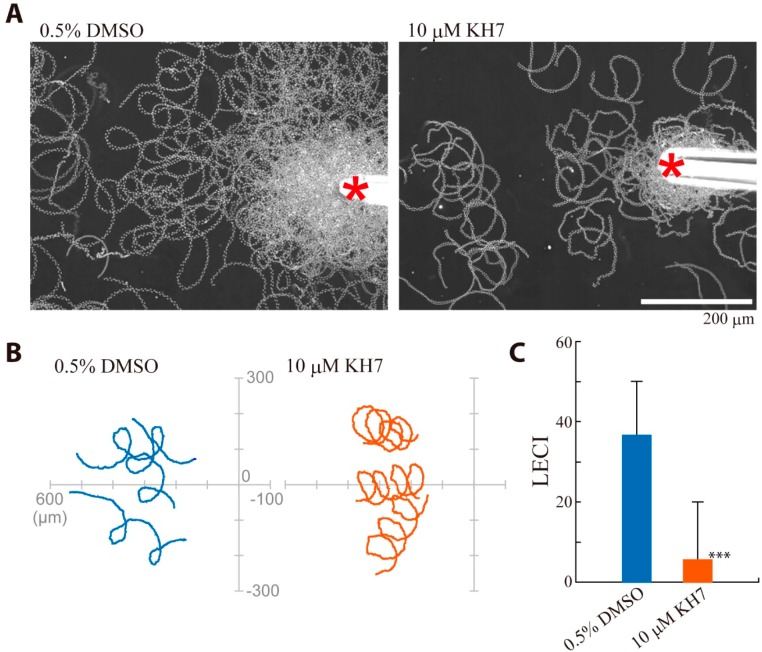
Effects of sAC inhibitor KH-7 on chemotaxis of *Ciona* sperm. (**A**) Swimming trajectories during 2 s of sperm treated with 0.5% DMSO (control, **left**) and sperm treated with 10 μM KH-7 (**right**). The photo was integrated from 200 images taken at 100 fps. The red asterisks represent the tip of glass capillary filled with SAAF; (**B**) Sperm path trajectories of three representative sperm are shown; and (**C**) Comparison of linear equation chemotaxis indices (LECI) showing significant inhibition of chemotaxis by KH-7. Values are means ± SE. *n* = 29 (control) and 34 (KH-7). *** Significant at *p* < 0.001 (Student’s *t*-test) as compared with the control.

**Figure 7:**
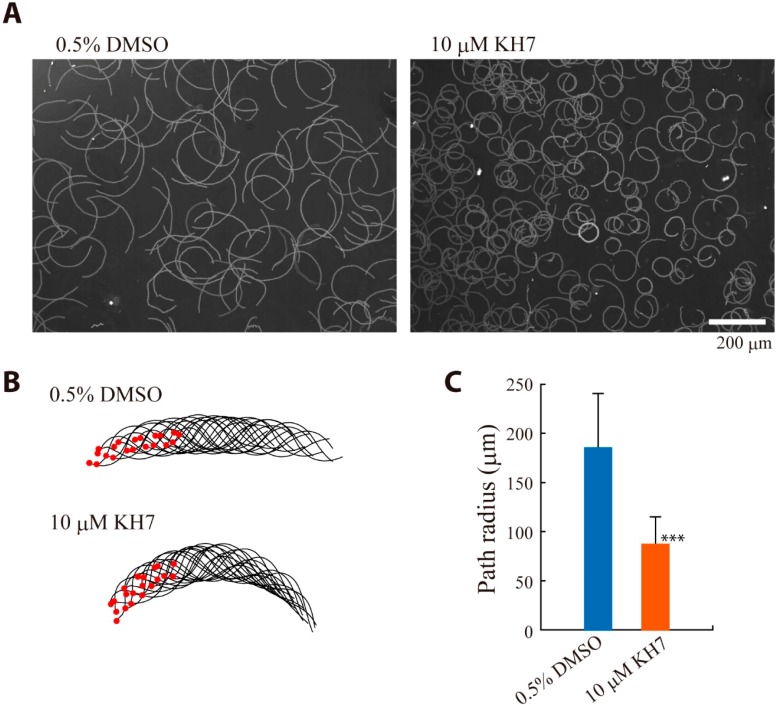
Effects of sAC inhibitor KH-7 on the swimming trajectory and flagellar wave asymmetry of *Ciona* sperm. (**A**) Swimming trajectories during 1 sec of sperm treated with 0.5% DMSO (control, **left**) and sperm treated with 10 μM KH-7 (**right**). Sperm were activated by 1 mM theophylline. The photo was integrated from 200 images taken at 200 fps; (**B**) Flagellar bending pattern of control or KH-7 treated sperm. Data from 20 waveforms are overwritten. Red dots indicate head position; and (**C**) Diameter of the circle of sperm trajectory in the presence of DMSO (control) or KH-7. Values are means ± SD. *n* = 120 (control) and 128 (KH-7). *** Significant at *p* < 0.001 (Student’s *t*-test) as compared with the control.

Sperm chemotaxis in *Ciona* is controlled by transient increase in intracellular Ca^2+^ concentration, followed by calaxin-mediated propagation of asymmetric flagella waveforms [34,35]. Next, we examined the effect of KH-7 on the chemotactic behavior (Figure 6). The movement with quick “turn” toward the attractant was significantly suppressed by KH-7 (Figure 6A,B). The calculated chemotactic index (LECI, [2]) demonstrates that sperm chemotaxis is significantly inhibited by KH-7 (Figure 6C). We found that diameter of the circle of sperm trajectory became smaller in the presence of KH-7. An analysis of flagellar waveform asymmetry indicates that flagellar waveform become more asymmetric in the presence of KH-7 (Figure 7). This suggests that intracellular Ca^2+^ concentration increases by KH-7, most likely by inhibiting Ca^2+^-efflux system, such as Na^+^/Ca^2+^ exchanger [36]. It was also reported in mammalian sperm that HCO_3_^−^-mediated sAC activity is involved in conversion into symmetrical flagellar waveform [27]. Alternatively, sAC could participate in the mechanism for the resumption from calaxin-mediated asymmetric waveform to symmetric waveform [35]. In either case, inhibition of sAC by KH-7 results in loss of efficient turn movement, which would cause suppression of chemotactic movement of sperm. It is still possible that the loss of efficient turn movement is secondarily resulted from KH-7-induced decrease in swimming velocity.

### 2.4. Effects of AC Inhibitors on cAMP-Dependent Protein Phosphorylation in Ciona Sperm

**Figure 8:**
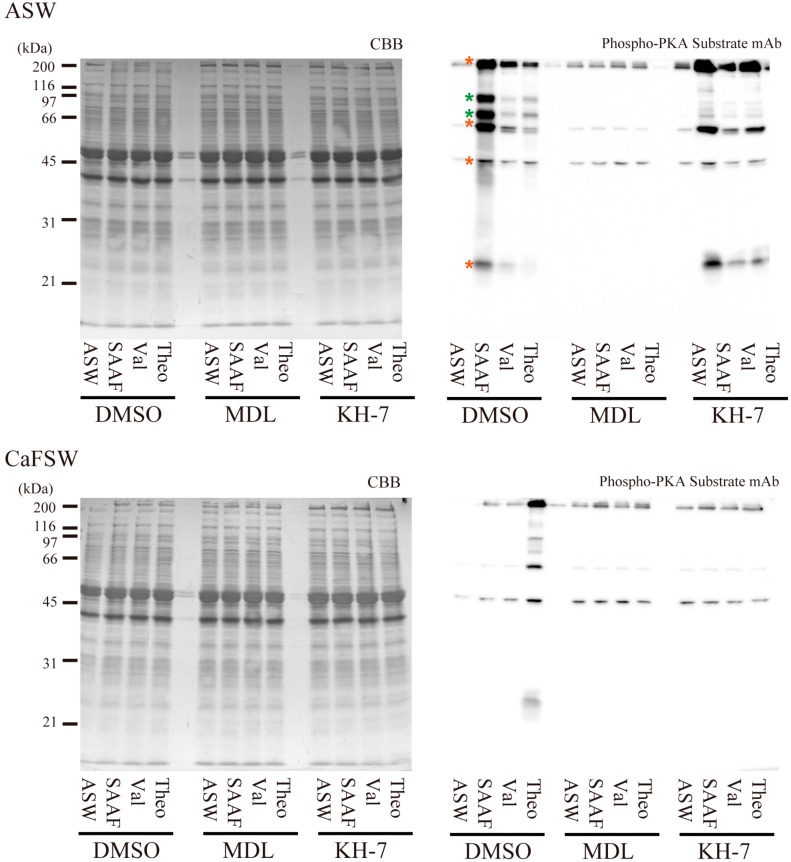
Effects of AC inhibitors on SAAF-, valinomycin (Val)- or theophylline (Theo)-induced protein phosphorylation by protein kinase (PKA). CBB-stained pattern and corresponding western blot with anti-PKA-substrate antibody were shown for sperm treated by artificial seawater (ASW), 100 nM SAAF, 10 nM valinomycin or 1 mM theophylline with 0.5% DMSO (control), 100 μM MDL12330A or 10 μM KH-7. Data obtained in ASW (**top**) and in CaFSW (**bottom**) are shown. The percentage of polyacrylamide in the separating gel is 10%. Proteins with significant increase in phosphorylation by SAAF-activation are indicated by asterisks in the lane of sperm sample activated by SAAF in ASW. Those showing decreased phospholylation by KH-7 in ASW are marked in green and others are in red. Data show a typical result from three independent experiments.

cAMP-dependent protein phosphorylation is prerequisite for activation of sperm flagellar motility [3,37,38,39]. Next we examined the effects of AC inhibitors on the PKA-dependent protein phosphorylation in *Ciona* sperm (Figure 8). SAAF induced PKA-mediated phosphorylation of six proteins, including a high molecular mass (HMM) protein (possibly dynein heavy chain), 105 kDa protein, 80 kDa protein, 65 kDa protein, 48 kDa protein (possibly PKA regulatory subunit), and 26 kDa protein (Figure 8, asterisks). Phosphorylation of these proteins, except for the 48 kDa protein, was greatly diminished in sperm treated with MDL12330A in both the presence and absence of Ca^2+^. Phosphorylation of both the 105 and 80 kDa proteins were suppressed by KH-7 in ASW, suggesting that these are closely related to the function of sAC. In contrast, HMM, 65 and 26 kDa proteins were significantly phosphorylated in KH-7-treated sperm, suggesting that these proteins are involved more in basic activation of sperm motility than in the increase of beat frequency or in chemotactic behavior (see motility experiments in Figure 4, Figure 5, Figure 6 and Figure 7). In CaFSW, only theophylline-treated sperm without AC inhibitors exhibit motility. The phosphorylation pattern well coincided with the motility data (Figure 4); phosphorylation of all the six major proteins of theophylline-treated sperm in CaFSW was more prominent than the others (Figure 8).

In the present study, we showed distinct roles of tmAC and sAC in the regulation of sperm motility in *Ciona*. tmAC is thought to be involved in the basal pathway for activation of motility; it transiently synthesizes high level of cAMP in response to SAAF-induced Ca^2+^ influx and membrane hyperpolarization, resulting in the activation of PKA that phosphorylates axonemal proteins for motility. In contrast, sAC is thought to mainly participate in the regulation of the asymmetry of flagellar waveforms and increase of beat frequency. The swimming velocity of SAAF-activated, KH-7-treated sperm showed half of the control sperm. This is also the case in sperm activated by valinomycin as well as theophylline-activated, KH-7-treated sperm. Thus, it is likely that flagellar motility is regulated by at least two pathways: SAAF-dependent tmAC pathway for basic activation for motility and SAAF-independent sAC pathway for full motility with high beat frequency and waveform conversion leading to sperm chemotaxis. Proteins that are differently phosphorylated with or without AC inhibitors may be the key to prove this model. Several studies demonstrate distinct localization of AC isoforms in mammalian [40,41,42] and sea urchin sperm [14]. Studies on immunolocalization of ACs in *Ciona* sperm would surely provide further evidence for the roles of ACs in the regulation of sperm motility.

## 3. Experimental Section

### 3.1. Ciona Sperm

The ascidian *Ciona intestinalis* was supplied by the Education and Research Center of Marine Bio-Resources, Tohoku University, Onagawa, Japan, and the National Bio-Resource Project of the Ministry of Education, Culture, Sports, Science and Technology (MEXT). Animals were kept in aquaria under constant light for accumulation of gametes without spontaneous spawning. Semen samples were collected by dissecting the sperm duct and kept on ice until use.

### 3.2. Chemicals and Solutions

Artificial seawater (ASW) contained 462.01 mM NaCl, 9.39 mM KCl, 10.81 mM CaCl_2_, 48.27 mM MgCl_2_, and 10 mM Hepes-NaOH (pH 7.6). Ca^2+^-free sea water (CaFSW) was as ASW without CaCl_2_ but with 478.2 mM NaCl and 10 mM EGTA. Low Ca^2+^ sea water (LCSW) was as ASW with 1 mM CaCl_2_ and 476.7 mM NaCl. MDL-12,330A from Calbiochem (San Diego, CA, USA), KH-7 from Chemical Diversity Research Institute (Khimki, Russia) and valinomycine and forskolin from Sigma-Aldrich (St. Louis, MO, USA) were dissolved in dimethyl sulfoxide (DMSO). Theophylline from Sigma-Aldrich was dissolved in ASW. Other reagents were of analytical grades. SAAF was partially purified from the egg-seawater of *C. intestinalis* according to Yoshida *et al.* [43].

### 3.3. Sequence Analysis

BLAST searches of mammalian AC sequences in the *C. intestinalis* genome database (http://ghost.zool.kyoto-u.ac.jp) identified four *AC* gene models (*Ci-sAC*: KH.C10.393, *Ci-AC5/*6: KH.C14.32, *Ci-AC8*: KyotoGrail2006.694.2.1, *Ci-AC*9: KH.C14.534). Phylogenetic tree was constructed by ClustalW2 (http://www.ebi.ac.uk/Tools/msa/ clustalw2/) with the aid of MEGA5 [44].

### 3.4. Reverse-Transcriptase (RT) PCR

Total RNA was extracted from the endostyle, gill, testis and ovary of *C. intestinalis* by using Isogen (Nippon gene, Toyama, Japan), separated by chloroform:isoamyl alcohol 24:1 (Sigma-Aldrich) and precipitated by isopropanol. cDNA was synthesized from the total RNA of each tissue by using SuperScript III reverse transcriptase (Invitrogen, Carlsbad, CA, USA). PCR was performed with Elongase^®^ Enzyme Mix (Invitrogen, Carlsbad, CA, USA) and specific primers as follows: Ci-sAC, 5'-GATATTGGGGATGTTCCACG-3' (forward) and 5'-GTGCGGGCCATCTTAATAGA-3' (reverse); Ci-AC5/6, 5'-TGAATGTGTCGCCGTTATGT-3' (forward) and 5'-CAACGTTTACCGTATTGCCC-3' (reverse); Ci-AC8, 5'-ATGCTAGCTCACCTTGCGTT-3' (forward) and 5'-ATCAGTGTGCCATCTTTCCC-3' (reverse); Ci-AC9, 5'-AACCCCACCGACAATTACAA-3' (forward) and 5'-ACAACGGTTTCGAACAGACC-3' (reverse). Amplifications were carried out for 35 cycles (94 °C for 1 min, 42 °C for 2 min and 68 °C for 2 min).

### 3.5. Assessment of Sperm Motility

Semen was suspended in 2000 volumes of ASW containing DMSO or inhibitors and incubated for 3 min. The sperm suspension was immediately placed on a glass slide coated with 1% BSA to avoid adhesion of sperm to the glass. The sperm movement was recorded at RT under a phase contrast microscope (BX51, Olympus, Tokyo, Japan) with a 10× objective (UPlan FLN, Olympus, Tokyo, Japan) and analyzed using Sperm Motility Analysis System (SMAS, DITECT Corporation, Tokyo, Japan). The percentage of motile sperm and curvilinear velocity were analyzed before, 1 and 5 min after the addition of SAAF, theophylline and valinomycin (final concentration; 100 nM, 1 mM and 10 nM respectively). Analysis of chemotactic behavior and flagellar waveform of sperm was performed by the method previously described [35]. Images were recorded with a high-speed CCD camera (HAS-D3, DITECT Corporation, Tokyo, Japan) and analyzed using Bohboh software (Bohboh Soft, Tokyo, Japan).

### 3.6. Measurement of cAMP

Levels of intracellular cAMP were assayed by using Amersham cAMP Biotrak Enzyme immunoassay (EIA) System (GE Healthcare, Buckinghamshire, UK). Semen was suspended in 2000 volumes of ASW containing DMSO (WAKO, Osaka, Japan) or inhibitors and incubated for 3 min, and SAAF (final concentration 100 nM) was added to the suspension. The sperm suspension before, 10 s, 1 min and 5 min after the addition of SAAF were transferred to a lysis buffer to stop the reaction. The lysate was centrifuged at 12,000× *g* for 10 min at RT and the supernatant was used for the measurement of cAMP.

### 3.7. Immunoblotting by Anti-Phospho-PKA Substrate Antibody

Semen was suspended in 100 volumes of ASW or CaFSW containing DMSO or inhibitors and incubated for 3 min, and SAAF, theophylline or valinomycin (final concentration; 100 nM, 1 mM or 10 nM respectively) were added to the suspension. After 1 min cells were solubilized in a sample buffer; 62.4 mM Tris-HCl, 2% SDS, 4% glycerol, 0.004% bromophenol-blue, pH 6.8 and boiled at 95 °C for 2 min. Proteins separated by SDS-PAGE were transferred to a polyvinylidene difluoride (PVDF) membrane and subjected to Western blotting with anti-phospho-cAMP-dependent protein kinase (PKA) substrate monoclonal antibody (#9624, Cell Signaling Technology, Beverly, MA, USA) as the primary antibody and horseradish peroxidase (HRP)-conjugated anti-rabbit IgG (Invitrogen, Carlsbad, CA, USA) as the secondary antibody. The immunoreactive bands were detected using ECL-prime (GE Healthcare, Buckinghamshire, UK).

## 4. Conclusions

Three tmAC (AC5/6, AC8 and AC9) and one sAC are encoded in the genome of the ascidian *Ciona intestinalis*. All of these *AC* genes are expressed in the testis. From the analyses of sperm motility using inhibitors for tmAC and sAC, we show that tmAC and sAC play distinct roles of in the regulation of sperm motility. tmAC transiently synthesizes high level of cAMP in response to SAAF-induced Ca^2+^ influx and membrane hyperpolarization, resulting in the activation of PKA that phosphorylates axonemal proteins for basal activation of sperm motility. In contrast, sAC mainly participates in the regulation of the asymmetry of flagellar waveforms and increase in beat frequency in a HCO_3_^−^/pH-dependent manner. Phosphorylation of 105 and 80 kDa proteins appears closely related to this process. Thus, flagellar motility is likely to be regulated by two pathways: (1) SAAF-dependent tmAC pathway for basic activation of motility and (2) SAAF-independent sAC pathway for full motility with high beat frequency and waveform conversion.
